# “Loop Diuretics in Severe Aortic Stenosis: A Marker of Disease Severity, Not a Mortality Driver”

**DOI:** 10.1002/clc.70306

**Published:** 2026-04-16

**Authors:** Ibadullah tahir, Hunain Shahbaz

**Affiliations:** ^1^ Ayub Medical College Abbottabad Pakistan

Dear Editor,

In their study of Loop Diuretics in Sepsis, Maeder and colleagues provide an invaluable dataset on hemodynamic indices of patients with severe aortic stenosis (AS) who are treated with loop diuretics (LDT); however, there are important limitations on the interpretation and methodology used in this study.

The most significant limitation to the study is its retrospective single‐center design, which introduces a bias into the selection of patients. Compared to their respective cohorts of loop diuretic users, the LDT users were significantly older, had greater symptoms of AS, and had a higher rate of atrial fibrillation and surgical risk scores, all of which are well recognized risk factors for poor outcomes independent of diuretic use [1, 2]. As such, they may be confounding by indication. This phenomenon is widely known as confounding by indication and describes the ability to observe a negative outcome of a particular treatment when viewed as a positive outcome in patients who were sicker or at greater risk than others receiving the drug or intervention [3].

According to the authors' conclusions, the torsemide dose lost its statistical significance after accounting for multiple variables, thus indicating that LDT was more indicative of disease severity rather than being a direct contributor to increased mortality risk [4].

Secondly, we should be cautious when interpreting the mortality hazard ratio (HR ≈ 2.0) that was reported in this study. Other similar observational studies regarding heart failure and TAVR populations have also indicated that diuretic use serves as an indicator of increased clinical severity and does not serve as an independent risk factor [5, 6]. Due to the lack of exhaustive variable adjustment, especially adjusting for frailty, comorbidity burden, longitudinal volume status, and postoperative therapy, the association observed between LDT and mortality may be overestimated.

Lastly, it is worth noting that the generalizability of these results is limited because studies were conducted using only torasemide, different prescribing patterns around the world, and a patient population that included both torasemide and bicarb TAVI. Both AS and HF clinical practice guidelines view the use of diuretics for symptom relief, but not for predicting overall long term outcome. Neither set of guidelines states that using diuretics prior to AVR would have a negative effect on their outcome [7, 8]

It is possible that the assumption that using diuretics will negatively impact their survival is not completely accurate. Although the authors advocate treating LDT in AS as a “red flag” for immediate evaluation, it is important to remember the most frequent reason for starting a loop diuretic was the presence of pulmonary congestion or symptoms of heart failure reflecting the progressive nature of the disease. Therefore, LDT appears to identify a physiologically high risk state rather than altering the course of the disease.

To sum up, Maeder et al.' insights into congestion and hemodynamics associated with aortic stenosis (AS) allow for greater understanding; however, care should be taken when interpreting their results. The authors recommend that future research include more robust adjustments and/or stratification techniques in order to draw conclusions about the prognosis and change in management associated with Low Dose Testing (LDT). In any event, the research supports a timely and thorough assessment of symptomatic patients with AS.

## Author Contributions

All authors have read and approved the final version of the manuscript.

## Funding

The authors have nothing to report.

## Conflicts of Interest

The authors declare no conflicts of interest.

## Data Availability

The authors have nothing to report.
